# Can You Help ChatGPT Get an “A” in Organic Chemistry? Teaching Effective Prompting of Large Language Models for Reaction Prediction

**DOI:** 10.1021/acs.jchemed.5c01712

**Published:** 2026-03-27

**Authors:** Elizabeth S. Thrall, Olivia M. Vanden Assem, Julia A. Schneider, Joshua Schrier, Sebastian Tassoti

**Affiliations:** † Department of Chemistry & Biochemistry, 5923 Fordham University, The Bronx, New York 10458, United States; ‡ Center for Chemistry Education, Institute of Chemistry, 27267 University of Graz, 8010 Graz, Austria

**Keywords:** Undergraduate, Organic Chemistry, Computer-Based Learning, Large Language Models, Generative AI, Prompting, Alkene Reactions

## Abstract

As generative artificial intelligence (AI) tools such as large language models (LLMs) become widespread, they are increasingly finding applications in chemical sciences. Although LLMs have achieved impressive performance in many chemistry tasks, optimal performance requires proper use, including appropriate prompting techniques. Chemistry students are not generally taught strategies for effective LLM usage, especially for nonwriting tasks. Here we report an activity that introduces organic chemistry students to the use of LLMs such as ChatGPT for predicting the outcome of chemical reactions, specifically the types of alkene addition reactions taught in introductory organic chemistry courses. This activity exposes students to molecular representations, digitization of chemical reactions, train-test splitting practices for evaluating performance, and generalizable LLM prompting strategies, namely, the Five “S” prompt-writing approach and in-context learning. We tested this activity with chemistry students in the USA and in Austria and evaluated the activity through anonymous pre- and postlab surveys. Survey data revealed that students felt that they achieved their learning goals and that the activity was enjoyable. As chemistry students will inevitably interact with LLMs in their future careers, it is important to teach best practices for the effective and critical use of these tools in the context of chemistry.

## Graphical abstract

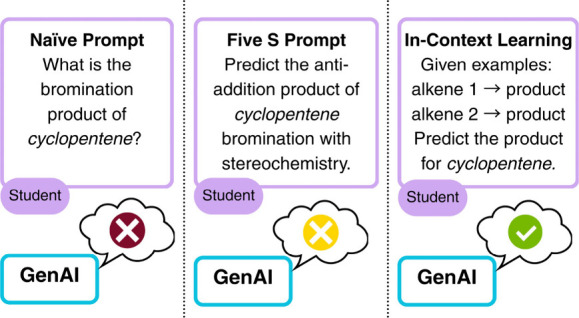

## Introduction

General purpose generative large language models (LLMs) are a powerful new artificial intelligence (AI) technology for advancing chemical research.^1–3^ Recent benchmarking studies have determined that the current generation of models (e.g., GPT-4o, Llama-3.1-405B, and Claude-3.5) exceeds human expert performance in answering chemistry-related questions.^4^ For organic chemistry, the latest generation of reasoning models (e.g., o3-mini) has shown marked advances in the ability to interpret molecular structures and make reaction predictions,^5^ although the best performance in organic synthesis prediction is still achieved by fine-tuning the models on chemical data.^6,7^

Students will inevitably use this type of technology professionally in the future,^8^ motivating the need to learn—and teach—its effective use. Recent editorials in this *Journal* ^9,10^ and reviews elsewhere^11–13^ have surveyed the limitations, applications, and open challenges related to the use of LLMs for chemistry education. Apart from studies into how GPT-3.5 answers mechanistic reasoning questions,^14,15^ however, ways to incorporate LLMs into organic chemistry education have not been discussed. Here, we demonstrate an activity for first semester undergraduate organic chemistry students, designed to be completed in 3 h, that teaches best practices for reliably using LLMs. The activity focuses on teaching general patterns for effective LLM prompting and the use of in-context learning, demonstrating these strategies as applied to organic reaction prediction, namely, alkene addition reactions.

## Purpose of the Activity

The main goals of this activity are to prompt students to:

- Assess task-dependent advantages of IUPAC and SMILES molecular representations.
- (Introduce and) practice general strategies for writing effective LLM prompts.
- (Introduce and) practice in-context learning methods for LLM prompting.
- Critically reflect on the effective use of LLMs for chemistry.

## Methods

### Molecular Representations

Organic chemistry coursework introduces students to various representations of molecular structure. The typical formats include text-representations in the form of IUPAC and common names, skeletal diagrams, Newman projections, and 3-dimensional models. Skeletal diagrams imply the notion of a *molecular graph*, in which vertices denote atoms and edges denote bonds. This representation emphasizes the molecule’s topological connectivity, rather than its 3-dimensional structure, although some aspects, such as stereochemistry, can be denoted using wedge notation. Molecular graphs can also be represented in machine-readable ways. The most common format is the simplified molecule-input line-entry system (SMILES), in which a molecule is represented as a text-based character string. Atoms are denoted by their atomic symbols, and bonds are typically implied by adjacency with single bonds often omitted. Double, triple, and aromatic bonds are represented by the symbols “=”, “#”, and “:”, respectively. Branching is indicated by parentheses, and ring closures are denoted by numbers following the atoms where the ring begins and ends. The notation supports chirality and isotopic specifications, making it a versatile tool for representing organic molecules.^16^ SMILES is a standard way to represent organic molecules for non-LLM machine learning tasks and has been used for this purpose in this *Journal*.^17,18^ Furthermore, all chemical structure drawing programs support import and export to the SMILES format, offering a user-friendly means of generating this representation.

### LLM Prompting

Effective prompting can make the difference between complete failure and perfect performance on chemistry problems.^19^ Qian recently reviewed prompting methods and their use in education.^20^ We adopt the Five “S” method for teaching chemistry students to write effective prompts,^21,22^ previously discussed by Tassoti in the context of an undergraduate "digital media for chemistry" course.^21^ This easily remembered framework (*Set the Scene/BeSpecific/Simplify your Language/Structure the Output/Share Feedback*) guides students to include key information in their prompt.

### In-Context Learning (ICL)

General purpose language models are trained on diverse sets of sources, and while they have good general chemical knowledge,^3–5^ these pretrained models often fail for very specific tasks such as reaction outcome prediction. One strategy to impart this expertise is to fine-tune the model parameters,^23^ which has been demonstrated to substantially improve both organic^6^ and inorganic^24^ synthesis prediction accuracy. However, this is computationally intensive and not approachable for the typical beginner. A surprisingly effective alternative is to merely provide a few relevant examples as part of the prompt text—a strategy referred to as few-shot in-context learning.^25^ (“Few-shot” because only a small number of examples are provided, in contrast to the “zero-shot” prompts where no examples are provided, and “in-context” because the examples are provided as part of the prompt input text, rather than as part of a separate training task.) This avoids the need to retrain the model, so it can be performed by any user as part of their prompt writing. We note that ICL is closely related to the strategy of retrieval-augmented generation (RAG) used to improve chatbot answer quality by including relevant reference material from an associated database.^26,27^ Additionally, from a pedagogical perspective, selecting appropriate examples requires students to identify relevant reaction classes and account for the possible creation of stereogenic centers. In a preliminary study, we found that providing 5–10 in-context examples sufficed to make accurate predictions using free, web-based LLM services (OpenAI GPT-4o, Google Gemini, and Meta Llama-3). Different reaction classes had varying performances; bromination reactions were predicted more accurately than hydroboration. In general, correct structural isomers were immediately predicted, but without suitable prompting, all of the models tested struggled with predicting correct stereochemical outcomes. A short overview of the types of prompts and expected LLM answers can be found in the Supporting Information for educators.

### Data Set and Model Selection

The strategies discussed above are generally applicable to any LLM and any problem, but for the sake of our demonstration, we chose alkene addition reactions typically covered in the first semester of organic chemistry. We sourced alkene reactions from the textbook used in each course: Smith’s *Organic Chemistry* ^28^ (Chapter 10) at Fordham and Clayden et al.’s *Organic Chemistry* ^29^ (Chapter 19) at University of Graz. However, any standard organic chemistry textbook should be adequate for this purpose. The students at both universities used OpenAI’s ChatGPT, accessed via a web interface; we chose this model both because many students are already familiar with it and because the University of Graz has its own ChatGPT interface, UniGPT. At Fordham, students started with the default model (ChatGPT 4o) but were eventually downgraded to 4o-mini after reaching the limit of free queries. At Graz, students used ChatGPT 4o, as well; however, one section used UniGPT, which also used the 4o model, due to a ChatGPT outage on the date of the activity.

### Institutional Context and Student Population

This activity was tested separately on undergraduate students at Fordham University (a private university in New York, USA) and University of Graz (a public university in Graz, Austria) enrolled in their first semester of organic chemistry. At Fordham, the exercise was implemented in an Organic Chemistry I Lab section (3 h block) with 18 students, all chemistry majors in their second-year (sophomore) fall semester. The activity was performed toward the end of the semester, after students had already been introduced to alkene reactions and the use of chemical drawing software, ChemDraw. Students worked in groups of four to digitize reactions and then worked on the prompting steps individually but collaboratively. At Fordham, students were permitted to opt out of data collection for this study, including the pre- and postlab surveys, but participation in the activity was required. In Graz, the exercise was implemented in a tutorial—a type of voluntary course in the Austrian university system where students can come to revisit content from a lecture, analogous to a recitation section in the American system, of the first Organic Chemistry Lecture course. Participation in the activity and data collection was optional at University of Graz. The class consisted of 30 students, all in the second semester of a chemistry bachelor’s degree program. The activity was offered three times, with students able to choose available times during weeks 10 or 11 of a 15-week semester, and performed in 3 h in lab groups of about 10. Students had been introduced to alkene reactions in the lecture course but did not have prior experience with ChemDraw. Therefore, all students completed the optional introduction to ChemDraw included in the lab handout before starting the main activity. Students worked in groups of three to digitize reactions and then worked on the prompts individually. After the initial results were obtained, students compared their prompts and then worked together to develop refined versions.

### Student Survey

To evaluate the activity and its effect on students’ perceived learning outcomes, we conducted anonymous prelab and postlab surveys. Students gave consent to researchers from both universities to analyze the data collected, and the necessary Institutional Review Board approval was obtained at Fordham (protocol no. 2820, classified as IRB Exempt). At the University of Graz, informed consent was given by the students, and they agreed to the use of the data for scientific purposes; the study was also exempt from review by the IRB. In the surveys, students were asked to indicate on a five-point Likert scale their agreement with a set of statements. The prelab survey consisted of a set of 10 statements, asking students to report their GenAI use (in terms of frequency, tasks, and prompting) in their chemistry classes and general university contexts. They completed the prelab survey approximately 1 week before the activity. In the postlab survey, students had to indicate their agreement with 28 statements. The first 10 were self-reported items toward the learning goals proposed for the activity. Five further questions were about their learning experience and whether they learned something about machine learning, GenAI, prompting, and organic chemistry. Three items from the prelab survey were repeated in the postlab survey. The survey was rounded out by 10 questions on the overall lab experience and the use of GenAI in the lab (challenges, confidence for future use, attitude toward GenAI, future learning about GenAI). In addition to these closed questions, 5 open-ended questions were asked to help improve the learning experience in an iterative process. The focus was on length, best and worst parts of the lab, additional information needed, and suggestions for improvements.

## Hazards

There are no hazards associated with this work.

## Activity Content

The student activity consists of four sections, as can be seen in Figure 1, along with an estimation of the time needed for all parts of the activity. The timeframes are to be seen as a suggestion and can vary depending on the student group.

**Figure 1:**
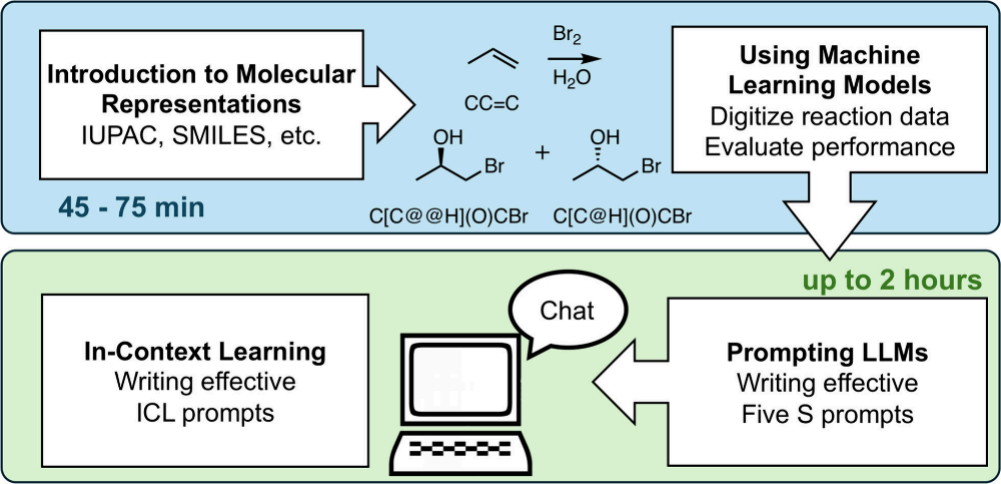
Overview of the activity and suggested time frames for the first and second parts, taken as estimations from implementations at both Graz and Fordham.

*Introduction to Molecular Representations* provides an overview of different graphical representations of molecules as well as stereochemistry and representations of chiral centers. The text introduces the use of machine-readable text strings to describe molecules: IUPAC names, SMILES strings, etc. Students are directed to a brief online introduction to SMILES by the US Environmental Protection Agency which provides basic information sufficient for describing alkanes/alkenes with simple substitutions.^16^ An optional activity introduces students to the use of ChemDraw to convert between molecular structures, IUPAC names, and SMILES representations.

### Using Machine Learning Models

*Train-Test Split* explains the machine-learning practice of defining distinct training and test sets. In the activity, student lab groups digitize a data set of ∼10 alkene reactions from one of three classes (hydroboration oxidation, halogenation, or halohydrin formation). Students are instructed to obtain the example reactions from their organic chemistry textbook and to use ChemDraw to get IUPAC names and molecular structures. In this activity, students are not training a new model or updating model parameters; instead, they apply the train/test concept by separating reactions included as in-context examples from reactions reserved for evaluation. *Generative AI and Large Language Models (LLMs)* provides a brief introduction to LLMs like ChatGPT.

*Prompting LLMs* introduces students to the Five “S” prompting framework for LLMs.^22^ In the activity, students first use this strategy for a nonchemistry example (asking the LLM “What does a dog say?”), comparing the outputs with a simple/naïve prompt to better prompts using the Five “S” strategy. Students then repeat this test of a simple prompt versus Five “S” prompt using examples taken from their data set of alkene reactions and compare the results.

*In-Context Learning* offers students the technique of few-shot in-context learning as an alternative to more costly or complex approaches like fine-tuning. In the activity, students first repeat the “What does a dog say?” example using the ICL approach to better constrain the desired output type. Students then test the ICL approach for their alkene reaction data. Students are instructed to use three training examples from their data set for the ICL prompt and to test the model predictions with other examples that are not in the training set, building upon the Train-Test split ideas introduced earlier.

*Reflection* invites students to self-assess their ability to write prompts and evaluate the reliability of the AI-generated answers. Instructors could expand this to include broader ethical and societal considerations of Gen AI or conduct a postactivity discussion.

## Instructor Observations, Development Process, and Evaluation

From an instructor’s perspective, this activity was broadly successful, although some refinements were needed after the initial trial at Fordham. In that implementation, students were largely able to complete the activity within the 3 h lab period; digitization of reactions took approximately 45 min, and the rest of the exercise took approximately 1.5–2 h. However, we identified several challenges. First, students found the activity to be a little too open-ended. They were confused about the end point; for example, they were unsure how many reactions they needed to test or whether ChatGPT needed to get the correct answer for all of their test cases. Additionally, the use of both the IUPAC name and SMILES led to confusion. Performance was somewhat better with IUPAC names than with SMILES (the inconsistent behavior of LLMs on different molecular representations is an active research question^30^), but some students mixed the two identifiers in their ICL prompts, which led to worse performance. We also realized that we had not explicitly addressed the reaction stereochemistry. Alkene addition reactions often lead to new stereogenic centers, but racemic mixtures of enantiomers are not always explicitly given in textbooks, and not all students had included such reactions in their training data sets for the ICL prompts. In addition to these conceptual challenges, there were several logistical issues. Students were using free ChatGPT accounts; after exceeding their limit of free queries, they were downgraded to a different model. Further, students struggled to find the best way to organize and share their digitized reaction data with other group members.

For the second implementation at the University of Graz, we revised the activity to address these issues. First, we provided more clarity on how many examples students should test and explicitly asked students whether the prediction was correct for those examples so that they understood that some incorrect predictions were expected. We changed the core activity to use IUPAC names exclusively; SMILES was included as an optional activity only. We explicitly addressed stereochemistry and instructed students to ensure that at least half of their examples included stereochemistry. Although model downgrading could have an impact on performance, we decided that the LLM landscape changes rapidly enough that it did not make sense to specify the use of a certain model; however, instructors should be aware of this potential issue. Finally, we created a spreadsheet template that students within a lab group could use to organize and share their digitized reactions; this spreadsheet could be accessed and updated simultaneously by multiple students using a collaborative online spreadsheet program such as Google Sheets.

During the refined implementation in Graz, the instructor carefully observed, focusing on the modified parts of the activity and the differences from the prior implementation at Fordham. One key difference was that students in Graz did not know ChemDraw yet and never learned explicitly about it in their studies. Thus, the ChemDraw introduction was implemented, and all students did this otherwise optional part of the activity. With this part averaging at 30 min, most groups finished the overall activity close to the 3 h mark. This introduction was mentioned as the most liked part of the lab by some students and was well received in general. Also, the revised introduction to stereochemistry and the use of SMILES as an optional feature were perceived positively. We wondered if students would struggle to work exclusively with IUPAC names; thus, a template was provided for scaffolding. To our surprise, students had no problems at all with the compound names, although the use of Excel was a bit of a struggle. A suggested modification for instructors seeking to implement the activity at their institutions is to set up a location on the cloud to share the data from the template with the entire class. Since some groups were slow when they created the training data set, they were asked to take data from other groups to remain within the time frame. Finally, the activity engendered unexpected discussion among the students and the classroom activity. A few groups initially had unsatisfactory results with the ICL prompt; this poor performance was used as an opportunity to reflect on the training data they produced. For example, when the training data consisted only of open chain alkenes, but the ICL prompt was tested on symmetrical rings, it led to incorrect stereochemistries. Instead of framing this as an error, we discussed the importance of selecting the training data carefully.

### Survey Results

A total of 18 students completed the lab at Fordham. One did not want to participate in the survey portion of the study, and another three did not fill out the whole survey and were thus discarded from the sample, arriving at *n* = 14 students at Fordham. In Graz, 30 students completed the activity, with one not giving consent to participate in the study but completing the activity, giving 29 students for the refined implementation. In the prelab survey (see the SI for data and statistical tests), we wanted to ensure that both groups were comparable in terms of prior GenAI use. Using a Mann–Whitney *U* test, a nonparametric statistical test which is suitable for ordinal data and smaller sample sizes, we found no statistically significant differences for 8 out of the 10 questions. Students at Fordham reported that they used GenAI less in general (*U* = 279, *z* = 1.970, *p* = 0.049) and less for chemistry-related applications (*U* = 309.5, *z* = 2.760, *p* = 0.006) than students in Graz, but overall, the GenAI use of both groups seemed to be very similar.

As discussed above, during the first implementation at Fordham some issues were spotted by the educators, and this was reflected in the students’ open-ended answers. Their comments strongly indicated that some aspects of the exercise could be clearer and more concrete; therefore, we revised the activity and evaluated whether the modifications improved student satisfaction. We found several improvements in student perception of the activity in Graz. First, students were more confident in GenAI and its answers and abilities. Also, they reported a more positive attitude: they were more confident in their own abilities regarding the use of GenAI, and they were more satisfied with the lab overall, agreeing it was a positive experience (see Table 1). Both groups of students tended to agree that GenAI use made the lab more entertaining and helped them achieve their learning goals, and they stated that they would like to see more AI tools integrated into other subjects and lab activities. They agreed relatively strongly that they encountered challenges when working with GenAI and also that they wanted to learn more about GenAI.

**Table 1:** Median Values (Mdn) and Interquartile Ranges (IQR) for the Five-Point Likert-Scale Answers as Given by the Students (1 = Fully Disagree, 5 = Fully Agree) in the Post-Survey^a^

	**Fordham**		**Graz**					
	**Mdn**	**IQR**	**Mdn**	**IQR**	***U*** ^a^	***z*** ^a^	***p*** ^a^	***r*** ^a^
I think that GenAI can solve complex chemistry problems.	2.5	3	4	1	321.5	3.071	0.002	0.468
I feel confident in the answers given by GenAI.	2	1	3	1	328	3.240	0.001	0.494
Using GenAI made this lab more complicated.	3	2	2	1	302.5	2.579	0.010	0.393
I encountered challenges while using the GenAI tool.	5	1	4	0.25	300.5	2.527	0.012	0.385
I feel more confident in my ability to use AI tools in future educational activities.	4	0.5	4	1	279.5	1.983	0.047	0.302
My attitude toward the use of AI in education has improved after this lab.	3	2.38	4	1.25	291	2.281	0.023	0.348
The AI tool exceeded my expectations in terms of usefulness.	2.5	2.5	4	1	279.5	1.983	0.047	0.302
The use of AI in this lab activity was a positive experience overall.	3	1	5	1	332.5	3.356	0.001	0.512
Overall, I enjoyed this lab.	4	1.5	5	1	305	2.644	0.008	0.403

a This table reports statistically significant differences. Please refer to the Supporting Information file that contains the raw data for an overview of all questions and answers across all students.

b For a Mann–Whitney *U* test, *U*-values, *z*-values, *p*-values, and effect sizes *r* are given.

While students’ perception of the activity improved after our revisions, statistical testing showed no difference in self-reported achievement of the learning goals of the activity. Students in the first round at Fordham and students in the second round in Graz both strongly agreed to achieving their learning goals in machine learning (Mdn = 5, IQR = 1), prompting GenAI with simple Five “S” strategies (Mdn = 5, IQR = 1) and with the more advanced ICL strategy (Mdn = 5, IQR = 1), and tended to agree on the statements about confidence in their ability to reflect on answers provided by GenAI (Mdn = 4, IQR = 1). This means that both groups of students thought that they had reached their learning goals, with the second and improved versions of the activity being a more enjoyable experience.

The students also answered three questions before and after the lab in a prepost format. There was a significant difference in their agreement with the statement “I can write effective prompts for GenAI.” (*U* = 1519, *z* = 5.135, *p* < 0.001) with a large effect size of *r* = 0.554 before (Mdn = 3) and after (Mdn = 4) the lab. There was no difference for the statements “I feel confident in the answers given by GenAI.” (*U* = 1111.5, *z* = 1.615, *p* = 0.106) and “I use GenAI answers as they are without checking them.” (*U* = 957.5, *z* = 0.285, *p* = 0.776). It should be noted that the authors did not expect these beliefs to change during a short intervention, while an increase in confidence in prompting abilities was expected. Thus, we cautiously conclude that after performing this activity, while students gained trust in their prompting abilities, they did not gain trust in GenAI tools, which could be a desirable way to teach about GenAI.

## Conclusion

We developed a new activity to introduce undergraduate organic chemistry students to the use of LLMs for organic reaction prediction, focusing on the specific example of alkene addition reactions. This activity exposes students to molecular representations, digitization of chemical reactions, training and testing data evaluation, and generalizable LLM prompting strategies. We tested this activity with students in the USA and Austria and used anonymous surveys to obtain feedback. Survey data indicate that students found this activity to be effective and enjoyable and that it improved their confidence in their ability to use AI tools. Students are increasingly interacting with LLMs in academic and nonacademic settings, and it is likely that they will continue to interact with AI tools in their future careers in chemistry or other fields. Thus, it is important to teach best practices for LLM use in the context of chemistry both now and as the field continues to evolve in the future.

## Supplementary Material













## Notes

SI file for students containing the instruction and learning goals for the activity (PDF, DOCX)
SI for instructors discussing examples of student prompts and corresponding LLM outputs (PDF, DOCX)
Spreadsheet file for instructors containing (1) a group sharing template for data set creation and (2) a solutions key for the template for bromination reactions (XLSX)
Raw data from the survey (XLSX)
