# Supplementary material for: Can You Help ChatGPT Get an “A” in Organic Chemistry? Teaching Effective Prompting of Large Language Models for Reaction Prediction
Source: J Chem Educ. 2026 Mar 27;103(4):2373–8. doi: 10.1021/acs.jchemed.5c01712 (PMC13085231; doi:10.1021/acs.jchemed.5c01712)
Supplement: Supplementary file 2 [file ed5c01712_si_002.docx]

**Supporting Information – Student Handout**

**Can you help ChatGPT get an “A” in organic chemistry? Teaching effective prompting of large language models for reaction prediction**

Elizabeth S. Thrall,^a,^* Olivia M. Vandem Assem,^a^ Julia A. Schneider,^a^ Joshua Schrier,^a^ Sebastian Tassoti^b,^*

*^a^*Department of Chemistry & Biochemistry, Fordham University, The Bronx, New York 10458, United States

*^b^*Center for Chemistry Education, Institute of Chemistry, University of Graz, 8010 Graz, Austria*

Corresponding Author E-Mail: [ethrall@fordham.edu](mailto:ethrall@fordham.edu) & [sebastian.tassoti@uni-graz.at](mailto:sebastian.tassoti@uni-graz.at)

Student Worksheet: Can you help ChatGPT get an “A” in organic chemistry?

# A. Project Goal

Can you teach a computer to predict the outcome of reactions of alkenes? Advanced large language models (LLMs), such as ChatGPT, Claude, Gemini, Llama, etc., provide a way to analyze vast datasets and identify patterns that may not be immediately apparent through traditional methods. In this lab activity, we will explore general strategies for using LLMs effectively and will apply those strategies to use LLMs to predict alkene reactivity. To enable us to use LLMs (or other computational tools) for chemistry, we will also discuss various ways that organic molecules can be represented on computers.

# B. Learning Goals

First, read through the statements below. This activity is designed to help you achieve these learning goals. The hope is that by the end of the activity, you will be able to agree with these statements!

**Molecular representations**

1. I know different types of molecular representation (skeletal diagrams, IUPAC names, SMILES strings).
2. I can convert between molecular representations using ChemDraw software.
3. I can choose a fitting representation depending on the advantages for the task I am facing (e.g. choose the best representation for a text-only document).

**Machine Learning**

1. I know what machine learning is.
2. I know why it is important to separate training from testing data.
3. I can apply my machine learning knowledge by creating a small chemical reaction dataset.

**Generative AI, LLMs and prompting**

1. I know the 5S prompting strategy as a general prompting strategy for effective large-language model (LLM) prompts.
2. I feel confident in using the 5S strategy for LLM prompting.

**In-context learning**

1. I know what in-context learning methods are in LLM prompting.
2. I feel confident in applying in-context learning methods in LLM prompting.

**Reflection of LLM use**

1. I can critically reflect on the prompts that I write for effective LLM use for chemistry.
2. I can critically reflect on the answers that I get from LLMs and adjust my prompting accordingly.

# C. Molecular Representations

| **In this chapter, my learning goals are:**   1. I know different types of molecular representation (skeletal diagrams, IUPAC names,   SMILES strings).   1. I can convert between molecular representations using ChemDraw software. 2. I can choose a fitting representation depending on the advantages for the task I am   facing (e.g. choose the best representation for a text-only document). |
| --- |

Before diving into machine learning applications, it is essential to understand the various ways that organic molecules can be represented on computers. Chemists represent organic molecules in different ways to focus attention on the composition, bonding, structure, and reactivity of the molecule, or to simplify. Understanding these formats will enable us to communicate chemical information effectively and use computational tools to their fullest potential. For concreteness, let’s consider the case of the **1-propene** molecule.

- The *chemical formula,* **C_3_H_6_**, tells us about the types and numbers of atoms present, but does not directly tell us anything about the structure or bonding of the molecule. This might be useful for computing molecular weights, but less useful if we want to know the molecular geometry.
-
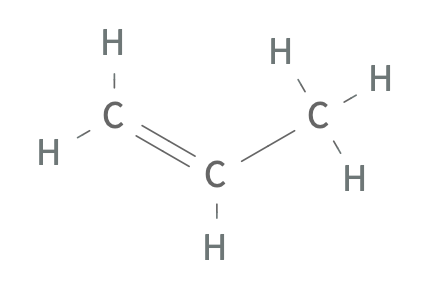
The *Lewis structure* shows all the atoms, bonds, and lone pairs of electrons in a molecule. This type of representation highlights the valence electrons and helps in understanding the molecule’s bonding and electron distribution. Typical depictions of Lewis structures only indirectly tell us about the 3-dimensional structure of the molecule. Additionally, Lewis structures are often very complicated to write for large molecules, and specifying all atoms, bonds, and lone pairs can obscure the features of interest.
- One way to simplify the representation is to use *skeletal diagrams* to represent the atoms and
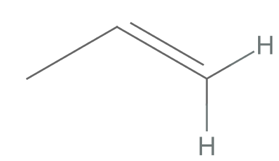
 bonds and their connections. In this representation, vertices denote atoms and edges denote bonds. A typical organic molecule is mostly carbon and hydrogen, so vertices without labels are carbons and hydrogen atoms are implicit—it is understood that every atom obeys the octet rule, so any carbon with less than four bonds should have the remaining bonds filled with hydrogen.

Skeletal diagrams may not include any hydrogens at all, so both of these diagrams are valid. Also notice how the only important aspect is the connectivity between the atoms; the left-right orientation is unimportant.
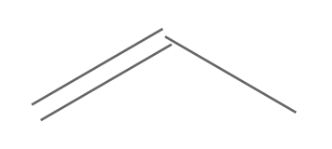


*Stereochemistry in molecular representations:*

*Stereochemistry* refers to the three-dimensional (3D) arrangement of atoms in a molecule. As you learned in your organic chemistry class, molecules with the same chemical formula and connectivity can have different 3D arrangements of atoms; these molecules are called *stereoisomers*. A molecule that cannot be superimposed on its mirror image is said to be *chiral*, and a chiral molecule can have more than one *stereocenter* where different stereochemistries are possible. Because stereoisomers can have very different properties, it is important to distinguish between them. In skeletal diagrams of molecules with chiral centers, solid wedges are used to indicate atoms in front of the plane of the page (or screen) and dashed wedges are used to indicate atoms behind the plane. Below is an example showing the two stereoisomers of alanine:

*Machine-readable molecular representation as a text-string*

Instead of drawing a structure, it is often more convenient to write the structure as text, particularly if we are using it as input to a computer program. We have already used two of these types of computer-friendly representations that consist only of text. We began by specifying the *IUPAC name,* **1-propene**. As you will recall, this is a systematic and standardized nomenclature used to uniquely identify chemical compounds based on their molecular structure, as defined by the International Union of Pure and Applied Chemistry (IUPAC). Often the full systematic IUPAC name can be quite unwieldy. For example, the systematic IUPAC name for sucrose (common table sugar) is (2R,3R,4S,5S,6R)-2-{[(2S,3S,4S,5R)-3,4-Dihydroxy-2,5-bis(hydroxymethyl)oxolan-2-yl]oxy}-6-(hydroxymethyl)oxane-3,4,5-triol. The R/S notation (2R, 4S, etc.) in this formula specifies the orientation of each stereocenter, which is generally assigned following the Cahn-Ingold-Prelog rules. (Review these conventions in your organic chemistry textbook, if needed.)

The *chemical formula*, C_3_H_6_*,* is also computer friendly, especially if we write it as **C3H6**—the subscripts are unnecessary to understand this representation. How might we incorporate information about the bonding of a molecule? *Condensed molecular formulas* provide a more compact way of writing the molecule's structure, showing the arrangement of atoms but omitting some or all of the bonds. The condensed molecular formula of 1-propene is **CH2=CH-CH3**. Notice how we only need to use characters on a computer keyboard (C, H, 2, 3, -, =) to represent the structure?

*SMILES (Simplified Molecular-Input Line-Entry System)* strings are a concise, linear notation system used to represent chemical structures in a text format that is both human-readable and machine-readable. Each SMILES string encodes the connectivity and stereochemistry of a molecule by specifying atoms, bonds, and branching patterns in a sequence of characters. This format is particularly useful for database searches, computational chemistry, and digital communication of molecular structures. Like the skeletal diagrams, hydrogen atoms are usually implicit in SMILES strings; additionally, we assume that a single bond between subsequent atoms is typical, and only need to denote double (=) and triple (#) bonds. For example, 1-propene is **C=CC**. Stated another way, every molecular graph represented in a skeletal diagram can be converted to a SMILES string and vice versa. In SMILES, chiral centers (stereocenters) are indicated using the symbols @ and @@. These symbols are used to denote the absolute configuration of the chiral center based on the Cahn-Ingold-Prelog priority rules. The @ symbol indicates clockwise (R) configuration, while @@ indicates counterclockwise (S) configuration when viewed from a specific direction. For double bond stereochemistry, SMILES uses the characters / and \ to indicate the geometry around double bonds. For example, C/C=C/C describes a molecule with *trans* configuration (E configuration), while C/C=C\C describes a molecule with *cis* configuration (Z configuration). In practice, it can be difficult to write SMILES by hand, and so it is typical to use molecular drawing tools, such as ChemDraw to generate the

Optional: If you are interested in the detailed rules, a good (short) introduction can be found online: <https://archive.epa.gov/med/med_archive_03/web/html/smiles.html>

*More information on other types of computer-readable molecular representation*

There are many other types of computer-readable molecular representations that *we will not use* in this exercise, but of which you should be aware. One especially notable example is the *InChI (International Chemical Identifier)* string*,* a format developed by IUPAC to encode detailed information about a molecule's structure, including connectivity, stereochemistry, isotopes, and electronic charge. This format enhances interoperability and data sharing across various chemical databases and software applications, facilitating accurate and consistent identification of chemical compounds. However, it is unwieldy for many common tasks. For example, the InChI string for 1-propene is **InChI=1S/C3H6/c1-3-2/h3H,1H2,2H3**, which is a bit of a mouthful! (Do you notice how it includes both the chemical formula, connectivity of the carbon atoms, and number of hydrogen atoms on each carbon?)

## Optional Activity: Computer-Readable Representation from ChemDraw

ChemDraw is a popular program that can be used to draw molecules and convert between different molecular representations. (Alternatively, you can use other programs like MarvinSketch or ChemDoodle.) Your instructor will show you how to use this software or provide training materials that you can review. If you already feel very confident with this type of software and are able to extract computer-readable molecular representations from ChemDraw, you can skip this activity. If you want a short overview of the software and how to use it for this task, please work on this activity. Complete the following exercises:

1. Consider the molecule: 2-methyl-3-ethylhexane
   1. Draw the skeletal form of this molecule on paper.
   2. Use ChemDraw to draw the molecule
   3. Copy and paste the IUPAC name of the molecule into ChemDraw to draw the molecule
   4. Copy-As SMILES and paste it into your notes.
   5. Briefly comment on how the branching structure is represented in the SMILES string.


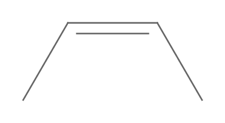


1. Consider the following molecule:
   1. What’s the IUPAC name of this molecule? (Try to figure it out without looking it up. Remember to specify the correct geometric isomerism.)
   2. Create the molecule in ChemDraw—you are free to draw it or specify it by name.
   3. Copy-As SMILES. What is different about this than the 1-propene example we considered above?
   4. Briefly comment on how geometric isomerism is represented and test your hypothesis by creating the SMILES string for the other geometric isomer of this molecule.
2. Now consider molecules with stereochemistry:
   1. Draw the two stereoisomers of alanine in ChemDraw.
   2. Copy and paste the IUPAC name of the two stereoisomers of alanine (see above, p. 3) into ChemDraw to draw them. Compare to your drawings to confirm that the structures agree.

Now, please rate how confident you are in your own abilities regarding your learning goals by ticking the corresponding box. **This is information for yourself and will not be seen and graded by your lecturers.** If you lack confidence, you can revisit the chapter or activities.

I know different types of molecular representation (skeletal diagrams, IUPAC names, SMILES).

Not confident at all ☐ ☐ ☐ ☐ ☐ Fully confident

I can convert between molecular representations using ChemDraw software.

Not confident at all ☐ ☐ ☐ ☐ ☐ Fully confident

I can choose a fitting representation depending on the advantages for the task I am facing (e.g., choose the best representation for a text-only document).

Not confident at all ☐ ☐ ☐ ☐ ☐ Fully confident

# D. Intro to Machine Learning: Train-Test Splits

| **In this chapter, my learning goals are:**   1. I know what machine learning is. 2. I know why it is important to separate training from testing data. 3. I can apply my machine learning knowledge by creating a small chemical reaction dataset. |
| --- |

*Machine learning* (ML) is a subdiscipline of artificial intelligence which focuses on algorithms that identify (“learn”) patterns and relationships within data using examples, rather than explicitly programmed rules. Machine learning is particularly powerful for tasks where we have many examples of what the correct answer *should* be, but we may not know how to program those rules manually. For example, to predict the outcomes of chemical reactions a machine learning model can be trained on a large dataset of past reactions, including the reactants, conditions, and products. By identifying patterns in this data, the model can learn to predict the products of new, previously unseen reactions, helping chemists to quickly and accurately forecast reaction outcomes without the need to explicitly encode complex chemical rules for each type of reaction.

There are typically three stages in developing a machine learning model. First, we need to collect the data. Second, we need to *train* the model by using some of those examples. Third, we need to *test* the model to see if it will be useful for new problems. The *train-test data split* should be designed in such a way that we can evaluate the model’s ability to generalize to new problems, rather than just memorize the answers it has seen in the training data. This is just like your experience as a student—it is more useful to learn about general rules of how reactions work, not just memorize thousands of answers to specific questions. Your examinations are designed with the same principle—if your instructor gave you exactly the same questions on both the practice tests and final examinations, then it would be impossible to determine if you knew the material or had merely memorized the answers.

In machine learning, a *train-test data split* is a method used to evaluate the performance of a model by dividing the dataset into two distinct subsets: the training set and the testing set. The training set is used to train the model, allowing it to learn patterns and relationships within the data. The testing set, on the other hand, is used to assess the model's performance on unseen data, ensuring that it can generalize well to new, unobserved examples. Consider an organic chemistry textbook filled with various problems about predicting the reactivity of alkenes. To apply machine learning, you could split the problems into a training set and a testing set. For instance, you might use the examples discussed in the main text of a chapter for training and the problems at the end of the chapter (and their answers) for testing. (Alternatively, you might combine all of the examples together and randomly select 80% of them for training and 20% of them for testing.) The main idea is that the training problems should be *representative* of the types of information that will be needed—containing various relevant molecular structures, reaction conditions, and the resulting products—but not contain any of the same examples that we want to test. Keeping the training and test data separate ensures that the model is not simply memorizing the training data but is instead learning to apply its knowledge to solve new problems.

## Activity: Creation of a Machine-Readable Dataset for an Alkene Reaction

1. Work with your lab partner(s) and use your organic chemistry textbook to create a dataset of alkene reactions in computer-readable format.
   1. Select one of the following types of **alkene reactions**: halogenation, hydroboration oxidation, or halohydrin formation reactions.
   2. Read through the relevant section of the textbook and collect the reactants and product(s) for each example of that type of reaction described in the chapter. Draw the reactants and products in ChemDraw and get their IUPAC names. Organize your group’s digitized reactions in the provided spreadsheet template, including the reactant and product IUPAC names and a ChemDraw image of the reaction. (Including an image of the reactions will make it easier for you to visualize the results, but DO NOT attempt to use these images as input for the LLMs.)
   3. After compiling all the solved examples from the textbook, read through the problems at the end of the chapter. Solve the problems and add them to your spreadsheet.
   4. **You will need a total of 10 unique reactions from steps b and c to train your machine learning model later in this activity.** It is important that at least half of these reactions include stereochemistry. If you can’t find enough reactions, ask the instructor for assistance.

Now, please rate how confident you are in your own abilities regarding your learning goals by ticking the corresponding box. **This is information for yourself and will not be seen and graded by your lecturers.** If you lack confidence, you can revisit the chapter or activities.

I know what machine learning is.

Not confident at all ☐ ☐ ☐ ☐ ☐ Fully confident

I know why it is important to separate training from testing data.

Not confident at all ☐ ☐ ☐ ☐ ☐ Fully confident

I can apply my machine learning knowledge by creating a small chemical reaction dataset.

Not confident at all ☐ ☐ ☐ ☐ ☐ Fully confident

# E. Generative AI and Large Language Models (LLMs)

Generative machine learning methods try to learn the underlying probabilities associated with data items; this in turn can be used to generate new items. Large language models (LLMs), such as ChatGPT, are a type of generative machine learning model which has been trained to predict the probability of a possible next word based on the words that have already been provided in a given text sequence. The underlying probability function has been trained on vast amounts of text data from the internet, which allows the model to predict and generate coherent and contextually appropriate responses. An essential concept in this process is the *context window*, which refers to the limited number of previous words the model considers when generating the next word. This context window is crucial for maintaining relevance and coherence in responses. Because any previous interactions in the context window are used, it is important to create new chat sessions to avoid leaking test data between your experiments.

**Supplementary Reading:** Stephen Wolfram “What is ChatGPT Doing…and why does it work?” (2023) <https://writings.stephenwolfram.com/2023/02/what-is-chatgpt-doing-and-why-does-it-work/>

# F. Prompting Large Language Models (LLMs)

| **In this chapter, my learning goals are:**   1. I know the 5S prompting strategy as a general prompting strategy for effective large-language model (LLM) prompts. 2. I feel confident in using the 5S strategy for LLM prompting. |
| --- |

Pre-trained *large language models* (LLMs), such as ChatGPT, are trained on vast amounts of text data to understand and generate human-like language. These models have already learned a wide range of patterns, syntax, and semantics from the training data, enabling them to perform various language tasks with impressive accuracy. However, LLMs are incapable of reading your mind. To use them effectively, you must express your problem and solution goals by writing a *prompt* to guide the model's responses effectively.

However, you cannot just write a prompt like you would write a Google search query— you need to consider the principles behind how LLMs work. There is no single recipe for writing a perfect prompt, and there certainly is no such thing as the perfect prompt you can just copy and paste. But there are a lot of strategies and frameworks that help you write a prompt. One such strategy is the 5S framework. We like this approach because its building blocks cater to the working principles of LLMs, and you can learn from each S instead of learning everything by heart. In a 5S prompt, you want to do the following:


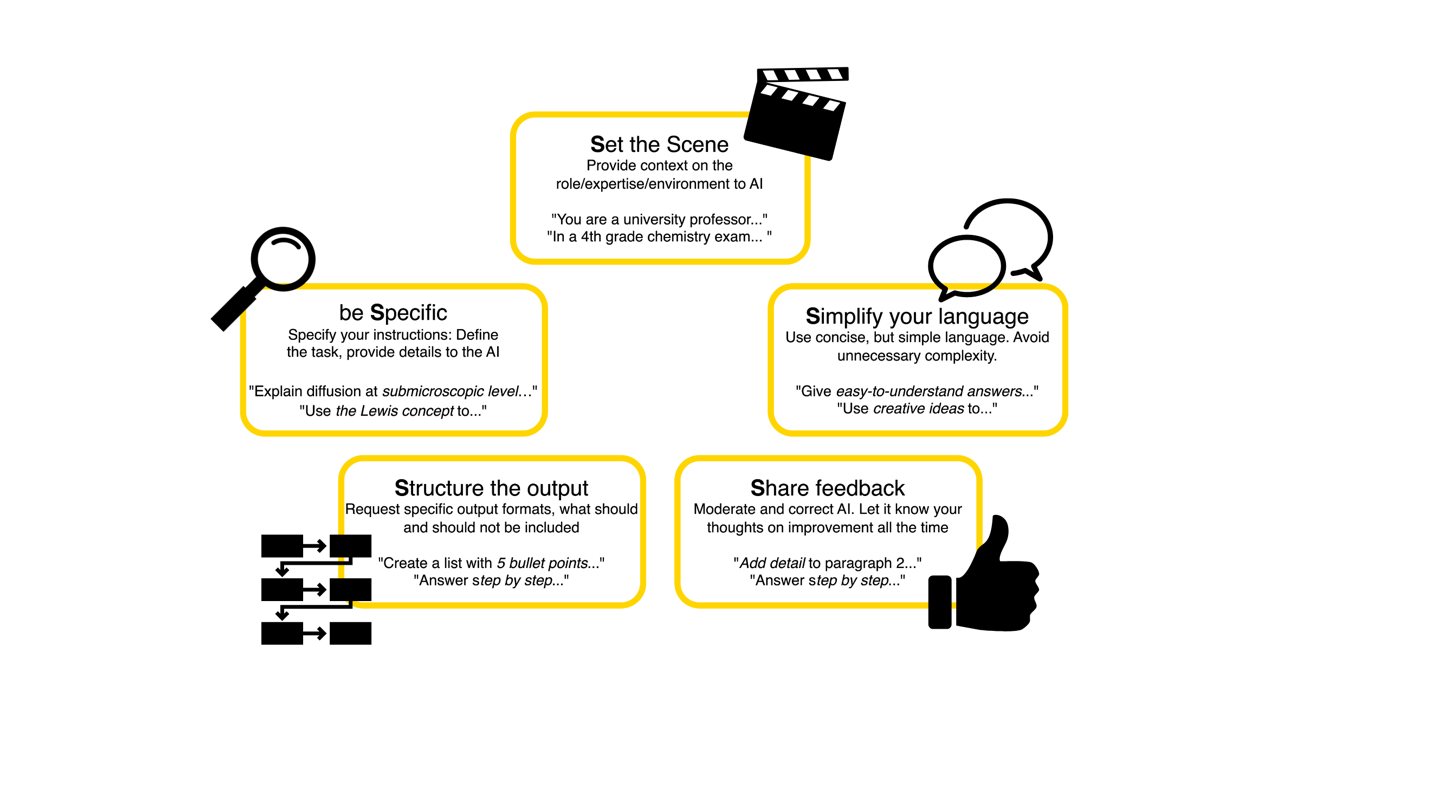


Reproduced from <https://doi.org/10.1021/acs.jchemed.4c01287>. Available under a CC-BY 4.0 license. Copyright Sebastian Tassoti.

***S****et the Scene* builds on the context sensitivity of generative AI. The more context you provide, the better a result potentially gets. You are trying to let it know what the context of your problem is by setting roles, expertise or an environment to the AI.

*Be* ***S****pecific* wants you to provide further context on what exactly the task is. You could specify the instruction by using relevant terms or additional information. Think about what exactly you want and specify it!

***S****implify your Language* is somewhat contradictory to being specific in a chemistry context, because you will need to introduce some technical terminology. Think about this as trying to keep to the minimum terminology, being clear and simple in telling the AI what you want it to do.

***S****tructure the Output* wants you to request a specific output format. This can be length (e.g., answer in one sentence), style (e.g., avoid complex terminology) and many other factors (try e.g., answer step by step—this might improve the output).

***S****hare Feedback* asks you to use your own prompt and the answer you get and to reflect on both. You can correct the LLM and work in the same context window or try your new prompt in a new context window. Modify your prompt and see if you can get better results!

The 5S framework should be a good starter to formulating better prompts for you. It is not meant as a “try everything at once” rigid guideline. Instead, start by working on one S, check the results and adapt your prompt by adding in more of the S’s step by step. You will see that it is not always possible to include all five S’s, but having 2-3 S’s in your prompts will usually help you. There is one thing to add—prompting strategies are situational. What works well for one problem might not be good elsewhere. So, you should focus on becoming flexible and proficient in your prompting instead of learning everything by heart and using phrases blindly.

## Activity: Writing Prompts Using the 5S Framework

1. Non-chemistry example: *What do dogs say?*
   1. Open your LLM program; your instructor will tell you if there is a preferred program or website for your course or at your university in general.
   2. Type in the prompt: “What does a dog say?” and evaluate it. The answer is probably a long explanatory text.
   3. But what if what you really want is just a single answer (like “Woof!” or “Wau wau” or “Guau”)? Use the 5S prompting approach to write a better prompt.
   4. Apply the prompt strategy you developed to produce the output for another animal (e.g., a cat, a pigeon, or a capybara).
2. Organic reaction example: *Predicting the outcome of organic reactions.*
   1. Open a new window in your LLM program. We are now going to use what we have learned to see if it can better predict organic reactions out of the box or with prompting strategies.
   2. Pick one of your training examples from Activity 3 and ask the LLM to predict the product formed. (Use the IUPAC representation for the input.) Don’t use any strategy—start with a prompt like “Predict the outcome of a [halogenation/hydroboration/...] reaction with [IUPAC name].” (Replace the brackets with one of your examples.)
   3. Now, open a new LLM window and write a prompt for the same reaction using the 5S style. Keep revising your 5S prompt until you are happy with the results.
   4. Compare your results with your lab partner(s). Test the “best” prompt that you collectively developed on three test examples each. (Pick different reactions and compile the results.) How often does the LLM get the correct answer?
   5. *Optional:* Open a new window in your LLM program. Use your best prompt for the same test examples as in step d, but this time use the SMILES abbreviation instead of the IUPAC name. Is the LLM performance better or worse with SMILES?

Now, please rate how confident you are in your own abilities regarding your learning goals by ticking the corresponding box. **This is information for yourself and will not be seen and graded by your lecturers.** If you lack confidence, you can revisit the chapter or activities.

I know the 5S prompting strategy as a general prompting strategy for effective large-language model (LLM) prompts.

Not confident at all ☐ ☐ ☐ ☐ ☐ Fully confident

I feel confident in applying this strategy to write effective prompts on a basic level.

Not confident at all ☐ ☐ ☐ ☐ ☐ Fully confident

# G. In-Context Learning

| **In this chapter, my learning goals are:**   1. I know what in-context learning methods are in LLM prompting. 2. I can apply in-context learning methods in LLM prompting. |
| --- |

The *pre-trained LLMs* used in the previous activity are sometimes called *foundation models* because they are trained on a diverse and extensive dataset to learn broad patterns and representations needed for general purpose tasks. While this provides an appropriate base for a wide variety of tasks, the lack of chemistry-specific training means that they may not possess the expertise needed for a highly specialized tasks, such as predicting chemical reactions, without additional training or contextual guidance. One way to do this is to *fine-tune* the model on relevant specialized task data. In fact, chemists have had great success in doing this for a variety of chemical prediction tasks (e.g., see <https://doi.org/10.1039/D4SC04401K>) but it can be technically challenging and getting the best results can require hundreds or thousands of examples.

However, a surprisingly effective alternative is to merely provide a few examples as part of the input prompt text—a strategy referred to as *few-shot in-context learning*. (Breaking this down: *few-shot* means that only a few examples are provided, and *in-context* means that they are provided as part of the prompt text rather than part of a separate training task.) In-context learning (ICL) avoids the need to retrain the model, making it easy for any user to include the relevant examples needed for the task at hand without the technical challenges of constructing a new model. For instance, in organic chemistry, if we want the model to predict the products of alkene reactions, we can provide a few examples of reactants, conditions, and products directly in the prompt. By showing the model these examples as part of the prompt, we effectively guide it to understand the type of task and the expected output without extensive retraining. This approach leverages the model's ability to generalize from just a few instances, making it highly efficient for specialized tasks like predicting chemical reactions or converting between different molecular representations, even with minimal specific data provided.

## Activity: Writing Prompts Using In-Context Learning

1. Non-chemistry example: *What do dogs say?*
   1. Open a new LLM window; this avoids having the previous information included in the context of the current evaluation.
   2. Type in the prompt:

What does a dog say?

Examples:

Cat -> meow

Bird -> chirp

and evaluate it. What do you get?

- 1. Apply the prompt strategy you developed to produce the output for another animal (e.g., a cat, a pigeon, or a capybara).

1. Chemistry example:
   1. We will now use ICL to predict reaction outcomes. Open a new LLM window.
   2. Let’s now write a prompt that uses ICL. For this, you have to:
      1. Provide your examples in a readable format. Use the IUPAC representation again and give ten unique examples:

Examples for [halogenation/...] reactions:

reactant -> product

cyclopentene -> (1S,2S)-2-bromocyclopentan-1-ol

[...]

- - 1. Give the LLM the specific task of predicting the outcome for a test reaction:

Give me the [halogenation/...] product of [reactant].

(Note: the test reaction must not be in the examples you provide!)

- 1. Working with your lab partner(s), test this approach on three test reactions each. (Pick different test reactions and compile the results.) Open a new context window for each test so that the LLM does not use information from the previous prompts. How often does the LLM get the correct answer?
  2. *Optional:* Open a new window in your LLM program. Use the same training and test examples as in step d, but this time use the SMILES abbreviation instead of the IUPAC name. Is the LLM performance better or worse with SMILES?

Now, please rate how confident you are in your own abilities regarding your learning goals by ticking the corresponding box. **This is information for yourself and will not be seen and graded by your lecturers.** If you lack confidence, you can revisit the chapter or activities.

I know what in-context learning methods are in LLM prompting.

Not confident at all ☐ ☐ ☐ ☐ ☐ Fully confident

I feel confident in applying in-context learning methods in LLM prompting.

Not confident at all ☐ ☐ ☐ ☐ ☐ Fully confident

# H. Reflection

After completing this lab, please rate how confident you are in your own abilities regarding your learning goals by ticking the corresponding box. **This is information for yourself and will not be seen and graded by your lecturers.**

I can critically reflect on the prompts I write for effective LLM use for chemistry.

Not confident at all ☐ ☐ ☐ ☐ ☐ Fully confident

I can critically reflect the answers I get from LLMs and adjust my prompting accordingly.

Not confident at all ☐ ☐ ☐ ☐ ☐ Fully confident
