# Supplementary material for: Can You Help ChatGPT Get an “A” in Organic Chemistry? Teaching Effective Prompting of Large Language Models for Reaction Prediction
Source: J Chem Educ. 2026 Mar 27;103(4):2373–8. doi: 10.1021/acs.jchemed.5c01712 (PMC13085231; doi:10.1021/acs.jchemed.5c01712)
Supplement: Supplementary file 3 [file ed5c01712_si_003.pdf]

Supporting Information - Instructors

Can you help ChatGPT get an 'A' in organic chemistry? Teaching effective prompting of large language models for reaction prediction

Elizabeth S. Thrall,[a,*] Olivia M. Vandem Assem,[a] Julia A. Schneider,[a] Joshua Schrier,[a] Sebastian Tassoti[b,*]

[a]Department of Chemistry & Biochemistry, Fordham University, The Bronx, New York 10458, United States

[b]Center for Chemistry Education, Institute of Chemistry, University of Graz, 8010 Graz, Austria*

Corresponding Author E-Mail: ethrall@fordham.edu & sebastian.tassoti@uni-graz.at

During this activity, the students will need to use GenAI-chatbots in several parts of their work. They will be instructed in two prompting techniques: using the 5S prompting framework for writing more intricate prompts and using in-context learning to get a retrieval-augmented generation out of the chatbot. To support instructors wishing to do this activity in their class and to give them some ideas on what to expect both from student prompting and GenAI performance, in the following we will give examples for different levels of prompts written by the students. They are taken directly from our intervention and include the GenAI chatbot answers. In Conversation S1, the students used no specific prompting technique to influence the output – they produced a zero-shot prompt. With such prompts, the answer is to be expected to be more general: the chatbot gives likely conditions like the solvent, gives some unstructured information using technical terms about the product and the course of the reaction, ending the answers with trying to create a Lewis structure, which it fails to do.

Conversation S1. Example conversation for naïve student prompts.

Student Prompt

Give me the halogenation product of cyclopentene with bromine

GenAI chatbot answer

Cyclopentene reacts with $\mathbf{Br_2}$ (in an inert solvent like CCl_4) by **electrophilic addition** across the double bond.
This forms a **vicinal dibromide** with **anti-addition** (via the bromonium ion intermediate).
Product: trans-1,2-dibromocyclopentane
(formed as a **racemic mixture** of enantiomers)
Structure (conceptually):

```
 Br     H
  \    /
   C-C
  /    \
 H      Br
```

So the answer is: **trans-1,2-dibromocyclopentane**.

Answer given by ChatGPT 5

Students were given a scaffold in the 5S prompting framework to improve their zero-shot prompts and increase relevance of the results generated. This framework was tested in previous courses and the prompts students wrote in this setting were comparable to our experiences. They managed to set the scene, be specific and structure the GenAI output as exemplified in Conversation S2. As seen in this example, the answers generated were comparable in content but included more detail (e.g. a description of the reaction mechanism). While both the zero-shot approach and the 5S prompt did work with simple unsubstituted cyclic and open-chain alkenes as seen in Conversations S1 and S2, more complex alkenes should be

expected to result in a higher rate of chatbot failure, especially when stereochemistry needs to be predicted precisely.

Conversation S2. Example conversation – prompt using 5S strategies.

Student Prompt

Pretend you are a organic chemist and want to know the outcome of the following reaction. Ethylene + bromine. You want to know the outcome because it is important for further research. Be specific and talk in a scientific way. As a result you want the reaction as a text and as a formular.

GenAI chatbot answer

As an organic chemist, investigating the outcome of the reaction between **ethylene (ethene)** and **bromine (Br_2)** is fundamental, especially when studying **electrophilic addition mechanisms**, testing for unsaturation, or designing halogenated intermediates for further synthesis. This reaction is one of the most classical and illustrative examples in organic chemistry.

📘 Reaction Overview (Textual Description)

Ethylene ($CH_2=CH_2$), a simple alkene, undergoes an **electrophilic addition reaction** with bromine (Br_2) in an inert solvent such as **CCl_4** or **CH_2Cl_2**. The reaction proceeds via the formation of a **cyclic bromonium ion** intermediate, which is then opened by a nucleophilic attack from the bromide ion (Br^-), resulting in the formation of **1,2-dibromoethane**.
This reaction is **stereospecific** and gives an **anti-addition** product due to the backside attack on the bromonium ion.

□ Reaction Formula

$CH_2=CH_2 + Br_2 \rightarrow BrCH_2–CH_2Br$
(or more specifically):
$C_2H_4 + Br_2 \rightarrow C_2H_4Br_2$
Product: **1,2-dibromoethane**

□ Reaction Mechanism (Steps in Brief)

1. **π Electrons Attack Br_2:** The π electrons of ethylene attack a bromine molecule, polarizing it.
2. **Formation of Bromonium Ion:** One Br forms a bond to both carbon atoms (three-membered ring).
3. **Nucleophilic Attack:** The Br^- (from Br_2) attacks the more substituted carbon from the opposite side, leading to anti-addition.
4. **Final Product:** A vicinal dibromide is formed (1,2-dibromoethane).

Answer given by ChatGPT 4o

As the final step of this activity, students create a dataset of addition reactions and provide those reactions to the GenAI chatbot in an in-context learning prompt. Conversation S3 shows such a prompt written by students. They provide the GenAI chatbot with their data and ask a specific question. The answer changes to only include the product as asked from the chatbot. This specific answer also shows which of the data the chatbot used to check its answer, namely the cyclic alkenes provided to it. This would be a great starting point for classroom discussion about requirements for training data in machine learning. If students manage to create a good dataset without errors, educators can expect the chatbot success rate to be reasonably high.

Conversation S3. Example conversation – prompt using ICL.

Student Prompt

Examples for bromine halogenation reactions:
Reactant (IUPAC) Reageant (IUPAC) Product (IUPAC)
cyclohexene bromine (1S,2S)-1,2-dibromocyclohexane

There are some more examples. The first two are the educts and the last one in each column is always the product.
2-methylprop-1-ene bromine 1,2-dibromo-2-methylpropane
(E)-but-1-en-1-ylbenzene bromine ((1S,2R)-1,2-dibromobutyl)benzene
3-methylbut-1-ene bromine (S)-1,2-dibromo-3-methylbutane
(E)-2,6-dimethylhept-3-ene bromine (3S,4R)-3,4-dibromo-2,6-dimethylheptane
cycloheptene bromine (1R,2R)-1,2-dibromocycloheptane
prop-1-en-2-ylbenzene bromine (R)-(1,2-dibromopropan-2-yl)benzene

Give me one halogenation product of cyclopentene. Use only the information provided above. I will work out the stereoisomer myself.

GenAI chatbot answer

Based on the patterns in the provided data, the **halogenation of cyclopentene with bromine** proceeds via:

- **Electrophilic anti-addition** of Br_2 across the double bond.
- Formation of a **vicinal dibromide** with defined stereochemistry (trans).
- Naming follows the IUPAC system with stereochemical descriptors.

Predicted Product:
(1R,2R)-1,2-dibromocyclopentane
This is consistent with entries like:

- cyclohexene → (1S,2S)-1,2-dibromocyclohexane
- cycloheptene → (1R,2R)-1,2-dibromocycloheptane

These show that for simple cycloalkenes, the bromine atoms add anti to each other, yielding **trans-1,2-dibromocycloalkanes** with the (1R,2R) or (1S,2S) configuration depending on absolute stereochemistry.

Answer given by ChatGPT 4o

In the later stages of the lab some students tested the limit of the GenAI-chatbots further. They discussed among themselves whether the chatbot would only take data from their prompt and tried to restrict it to the data they gave to it. To test their hypothesis, they tried to trick the chatbot into brominating benzene by naming it by its full IUPAC-name with the double-bonds listed separately as can be seen in Conversation S4. This was a great moment for discussion about ICL prompts and their limits.

Conversation S4. Example conversation – signs of reflected student behavior in trying to trick the LLM based on ML principles they had learned.

Student Prompt

Give me the halogenation product of 1,3,5-Cyclotrihexene. Use only the information provided above.

GenAI chatbot answer

Using only the information provided in your previous examples, we can infer the halogenation product of **1,3,5-cyclohexatriene** (also known as **benzene**) with **bromine**.

Key Observations from Your Data:

- All **alkenes** undergo **anti-addition** of Br_2 across **a single double bond**, forming **vicinal dibromides**.
- However, **conjugated systems or aromatic rings** (like benzene) are **not** represented among your examples.
- The name **1,3,5-cyclohexatriene** suggests a **non-aromatic view**, but chemically, it *is* benzene — an **aromatic compound**.

Based Strictly on Provided Data:

Since there is **no example** involving **aromatic bromination** or **electrophilic aromatic substitution** in the provided data, and since all products are vicinal dibromides from **alkene addition reactions**, we **must exclude aromatic reactivity** from consideration.

That said, there is **no basis in your data set** to suggest that **1,3,5-cyclohexatriene** undergoes a simple bromine **anti-addition**. Therefore:

Answer:

⚠️ **No halogenation product can be predicted for 1,3,5-cyclohexatriene using only the information provided above**, because your dataset includes only non-aromatic alkene halogenations and no examples of aromatic substitution.

Let me know if you'd like to expand the dataset to include aromatic reactions like electrophilic aromatic substitution (EAS).

Answer given by ChatGPT 4o
